# Uptake of community-based differentiated antiretroviral therapy service delivery and associated factors among people living with HIV in Ethiopia: a multicenter cross-sectional study

**DOI:** 10.3389/fpubh.2024.1390538

**Published:** 2024-08-08

**Authors:** Fasika Merid, Temesgen Mohammed Toma, Abraham Anbesie, Tamirat Gezahegn Guyo

**Affiliations:** ^1^Department of Public Health, Arba Minch College of Health Sciences, Arba Minch, Ethiopia; ^2^Department of Public Health Emergency Management, South Ethiopia Region Public Health Institute, Jinka, Ethiopia

**Keywords:** community-based DSD models, uptake, factors associated, PLHIV, CAG, PCAD

## Abstract

**Background:**

Achieving the 95–95–95 targets require an efficient and innovative person-centered approach, specifically community-based differentiated service delivery (DSD), to improve access to human immunodeficiency virus (HIV) services and reduce burdens on the health system. Therefore, this study aimed to assess the uptake of community-based DSD models and associated factors among people living with HIV (PLHIV).

**Methods:**

A multicenter cross-sectional study was conducted among PLHIV in public health facilities in South Ethiopia. Data were collected and entered into EpiData version 3.1 before being exported to Stata version 14 for further analysis. In the bivariable logistic regression analysis, variables with a *p*-value of ≤0.25 were included in the multivariable logistic regression analysis. A *p*-value of <0.05 was used to identify statistically significant factors.

**Results:**

Among 381 stable PLHIV, 55.91% were women. The median age (interquartile range) was 40 years (27–53). The uptake of community-based DSD models was 19.16%. Residence and disclosure were the two independent factors significantly associated with the uptake of community-based DSD models.

**Conclusion:**

One out of five stable PLHIV on antiretroviral therapy uptake the community-based DSD models. Improvement in uptake is needed in Ethiopia's resource-limited healthcare system to better achieve the 95-95-95 targets.

## Introduction

Globally, by the year 2022, ~39 million people were living with HIV, of which 29.8 million had access to life-saving antiretroviral therapy (ART), and ~630,000 died from acquired immunodeficiency syndrome (AIDS)-related illnesses (1). Africa is among the primarily affected continents by HIV, with 20.8 million people living with the virus and 260,000 deaths due to AIDS-related illnesses in the southern and eastern regions of the continent (1). Ethiopia is one of the sub-Saharan African countries with 610,350 people living with HIV (PLHIV) (2, 3). The prevalence of HIV in the country was higher (2.9%) in urban areas (4). There were ~11,000 AIDS-related mortalities in Ethiopia (2).

The United States Agency for International Development (USAID) offers differentiated service delivery (DSD) to improve care retention and address barriers to HIV treatment. The DSD models cater to unique population needs, focusing on client-centered care. Options include multi-month drug dispensing and decentralized drug distribution, reducing healthcare visits, and allowing clients to pick up drugs at home (5). Differentiated HIV care and treatment involves strategic modifications to client flow, schedules, and location of services to improve access, coverage, and quality of care for specific HIV subgroups (6, 7). This approach has the potential to overcome obstacles that clients face in adhering to medication schedules and visits (7).

Ethiopia implements less-intensive and more-intensive HIV treatment DSD models for established HIV patients. Less-intensive models include facility- and community-based approaches, while more-intensive models are implemented at the health facility level for those with advanced HIV disease, adolescents, key populations, and maternal and child health (8). Community-based differentiated service delivery (C-DSD) is a person-centered approach to improving access to HIV services and reducing burdens on the health system. It includes the Health Extension Professional-Managed Community ART Group (CAG) and Peer-Led ART Distribution (PCAD) (8, 9). These models were piloted in some areas of Ethiopia in 2019 but are currently under-implemented throughout the country (8).

Evidence from the studies conducted in Uganda (10–13), Malawi (14), and South Africa (15, 16) showed that the uptake of differentiated community ART models, including community client-led ART delivery (CCLAD), CAG, and Central Chronic Medication Dispensing and Distribution (CCMDD), ranged from 6 to 55%. A report from the evaluation of the DSD model in Kenya revealed that the overall uptake of the DSD model increased from 53% in 2018 to 85% in 2019 (17). In addition, a prospective comparative analysis conducted on client preference and viral suppression among PLHIV enrolled in the DSD model in Ethiopia showed that 59% of the PLHIV enrolled in the community DSD model preferred PCAD, while 41% preferred CAG (9).

In 2022, the global progress toward the 95–95–95 targets for testing, treatment, and viral load suppression showed that 86% of PLHIV know their HIV status, 89% of people who know their HIV status are on treatment, and 93% of PLHIV on treatment have suppressed viral loads (18). Ethiopia has nationally achieved the second and third 95 targets. A total of 84% of PLHIV know their status; 98% are on ART, and 98% of PLHIV on ART are virally suppressed (19). To achieve the 95–95–95 targets and to ensure that PLHIV are aware of their status, receive and maintain ART, and achieve viral suppression, community-based and health facility HIV service delivery points must provide effective, efficient, and high-quality services (20).

To achieve the 95–95–95 ambitious targets of UNAIDS, adopting efficient and innovative mechanisms for providing HIV treatment, care, and prevention services that meet the needs of various types of clients is essential (21). Community ART models aim to enhance patients' quality of services, optimize national ART program effectiveness, and minimize the need for clinic visits and system and patient difficulties (22–24). Community ART model uptake had a significant effect on removing obstacles to accessing care (25), reducing mortality, and reducing loss to follow-up (LFTU) (26). In addition, the uptake of community ART models can also improve retention in care (27) and contribute to money savings, which makes it a cost-effective intervention and reduces healthcare workforce requirements compared to individual care provision (28). In addition to the abovementioned benefits, a qualitative study conducted in three African countries, South Africa, Uganda, and Zimbabwe, reported that community ART models can enhance time-saving, support adherence, improve peer support, and reduce stigma (22). In Africa, community-based ART delivery had the potential to improve HIV care engagement, and outcomes related to ART in terms of adherence to ART, viral suppression, retention in care, and ART uptake were good among key populations (29).

Previous studies conducted on the uptake of different community-based DSD models showed that some sociodemographic and clinical factors like age, marital status, educational status, occupational status, duration on ART, and missed clinical appointment were associated with the uptake of community-based DSD models (10, 15, 16).

Expanding ART access to community-based ART service delivery programs resulted in remarkable achievement in poor resource settings (30). However, with the implementation of the community-based DSD model in Ethiopia, there is a lack of evidence regarding the uptake of the community-based DSD models and responsible factors, and no study was conducted in the study settings. Therefore, this study aimed to assess the uptake of community-based DSD models and associated factors among PLHIV in South Ethiopia.

## Methods and materials

### Study setting and period

The study was conducted at public health hospitals of Wolaita Sodo University Comprehensive Specialized Hospital, Arba Minch General Hospital, and Jinka General Hospital from June to September 2023. The hospitals are located in the administrative cities of the South Ethiopia Region, Wolaita Sodo, Arba Minch, and Jinka.

### Study design and participants

A facility-based, multicenter cross-sectional study was conducted among stable adult patients on ART for at least 1 year. Eligible participants include those with no adverse drug reactions requiring regular monitoring; a good understanding of lifelong adherence; evidence of treatment success (i.e., two consecutive VL measurements < 1,000 copies/ml, rising CD4 cell counts, or CD4 counts above 200 cells/mm^3^); no acute illness; those who were not pregnant or breastfeeding (4), and those who visited ART clinics in selected public hospitals during the data collection period.

#### Eligibility criteria

The inclusion criteria were stable HIV clients on ART who were at least 18 years old. Those who provided incomplete information from other health facilities were excluded from the study.

### Sample size determination and sampling technique

A single population proportion formula with a 50% prevalence, a 95% confidence interval, and a 5% margin of error was used to calculate the sample size, resulting in 384 participants. Since the total population was less than 10,000, a correction formula was applied, yielding a sample size of 350. Adding 10% to account for the non-response rate resulted in a final sample size of 385. The sample size was proportionally allocated among the selected public health hospitals, and a systematic sampling technique was used to select study participants.

### Data collection method

A predetermined structured questionnaire was used, and the data collection tool consisted of sociodemographic, behavioral-related, health service delivery-related, clinical, and treatment-related characteristics. An interview and medical chart observation of clients were used to collect the data. The client records were reviewed manually. Six BSc nurses collected the data, and three public health professionals supervised the data collection process.

### Study variable

The dependent variable was the uptake of community-based DSD models. Independent variables were sociodemographic characteristics (age, sex, residence, marital status, educational status, occupation, and monthly income), behavioral-related and health service delivery-related characteristics (khat chewing, alcohol use, frequency of condom use, and number of the sexual partner, facility type, ART facility catchment, and distance), and clinical and treatment characteristics (duration on ART, WHO clinical stage, viral load, CD4 count, regimen change, missed clinical appointment, disclosure status, history of tuberculosis infection, and social support).

### Data quality assurance

Before the data collection process, a pretest was conducted on 5% of the sample size, and 2 days of training were provided to data collectors and supervisors. The supervisors checked the completeness and consistency of the questionnaire filled out daily, and any necessary corrections were made.

### Data processing and analysis

The collected data were entered using Epi-Data version 3.1 and exported to Stata version 14 for further analysis. A descriptive statistical analysis was conducted, including frequency, percentage, median, and interquartile range. A bivariable logistic regression analysis was conducted to examine the relationship between the uptake of the community-based DSD models and independent variables such as age, sex, residence, marital status, educational status, occupation, monthly income, khat chewing, alcohol use, frequency of condom use, number of the sexual partner, facility type, ART facility catchment, distance, duration on ART, WHO clinical stage, viral load, CD4 count, regimen change, missed clinical appointment, disclosure status, history of tuberculosis infection, and social support. Variables with *p*-values of ≤ 0.25 were considered candidates for the multivariable logistic regression analysis.

The backward likelihood ratio was used to build the model. In the multivariable analysis, variables with a *p*-value of < 0.05 and an adjusted odds ratio (AOR) with a 95% CI were considered statistically significant. Multicolinearity and model adequacy were assessed using the variance inflation factor (mean VIF = 1.00) and the Hosmer and Lemeshow goodness of fit test (prob > chi^2^ = 0.5708).

## Results

**Figure 1:**
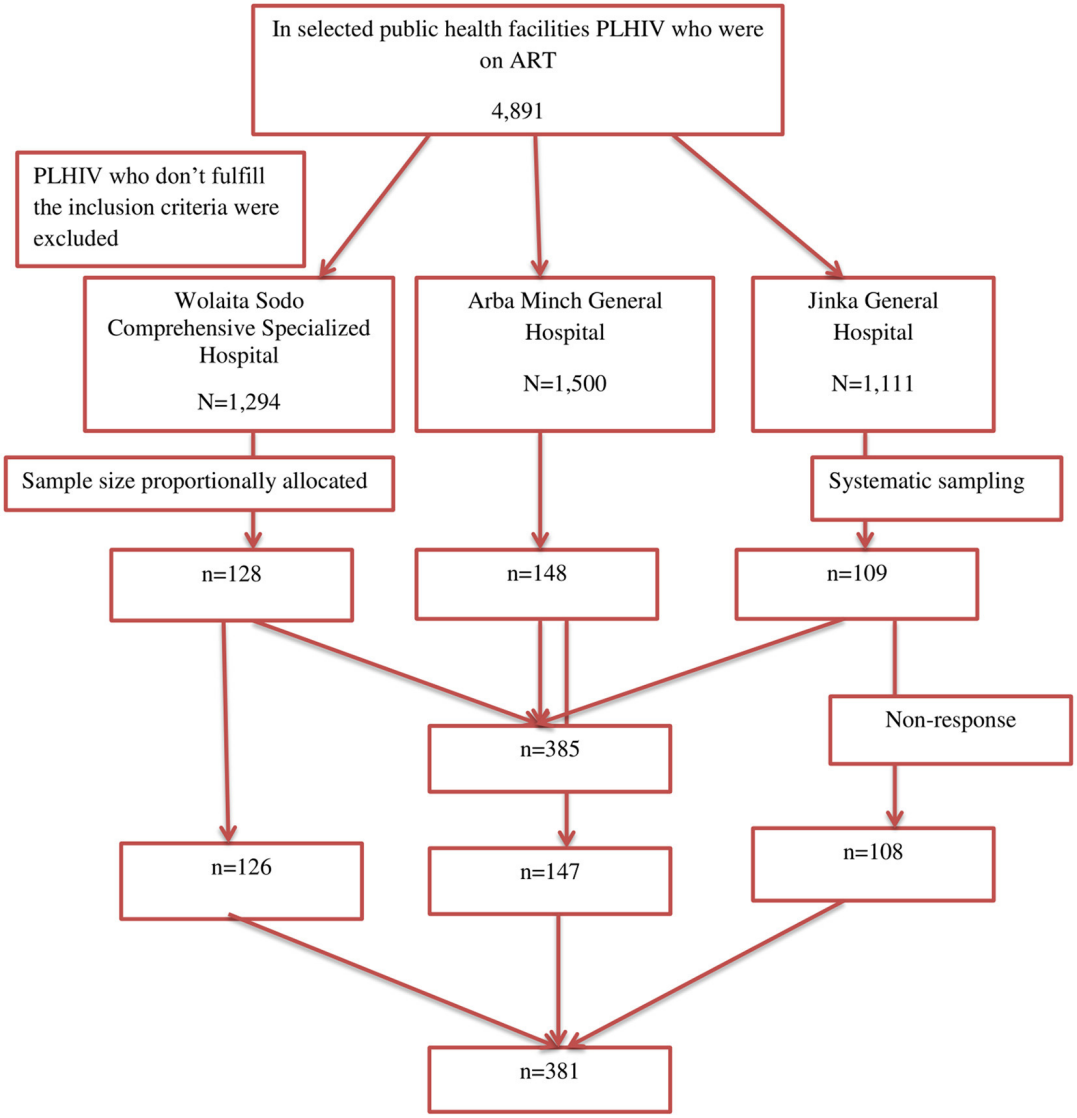
A flow diagram of the included study participants.

Among the total sample size, 381 participants were included in the analysis, with a response rate of 98.96% (Figure 1).

### Sociodemographic, behavioral-related, health service delivery-related, and clinical characteristics

The median age of the study participants was 40 years, with an interquartile range of 27–53 years. The majority of the participants, 255 of them (66.93%), were older than 35 years. More than half (55.91%) of the participants were women, and three-fourths of the participants (75.85%) were urban residents. Among 381 study participants, one-fourth of the participants (93 participants, 24.41%) had no formal education. Nearly one-fifth of the participants (16.8%) had alcohol use problems. Moreover, 24 participants (6.3%) had a history of TB co-infection, and 361 participants (94.75%) had undetectable viral loads. The baseline regimen was changed for the majority (87.93%) of the study participants. Additionally, one-fifth of the participants (19.69%) missed their clinical appointments, and 345 participants (90.55%) disclosed their serostatus (Table 1).

**Table 1:** Sociodemographic, behavioral-related, health service delivery-related, and clinical characteristics of PLHIV in public health facilities in South Ethiopia, 2023 (*n* = 381).

**Variables**	**Categories**	***n* (%)**
Age (in years)	≤ 25	27 (7.09)
	26–35	99 (25.98)
	>35	255 (66.93)
Sex	Male	168 (44.09)
	Female	213 (55.91)
Residence	Urban	289 (75.85)
	Rural	92 (24.15)
Marital status	Single	29 (7.61)
	Married	222 (58.27)
	Divorced/separated	65 (17.06)
	Widowed	65 (17.06)
Education	No formal	93 (24.41)
	Primary	142 (37.27)
	Secondary	97 (25.46)
	Tertiary	49 (12.86)
Occupation	Daily laborer	63 (16.54)
	Merchant	83 (21.78)
	Government employee	82 (21.52)
	Housewife	86 (22.57)
	Farmer/student	39 (10.24)
	Other^*^	28 (7.35)
Monthly income (Ethiopian Birr)	≤ 5,000	341 (89.50)
	>5,000	40 (10.50)
Facilities type	General hospital	255 (66.93)
	Comprehensive specialized hospital	126 (33.07)
ART facility catchment	Within the catchment	296 (77.69)
	Out of the catchment	85 (22.31)
Distance (in minutes)	≤ 10	6 (1.57)
	11–50	236 (61.94)
	>50	139 (36.48)
kchat chew	Yes	72 (18.90)
	No	309 (81.10)
Alcohol use problem	Yes	64 (16.80)
	No	317 (83.20)
Condom use	Always	57 (14.96)
	Sometimes	127 (33.33)
	Never	197 (51.71)
Number of sexual partners	No sexual partner	149 (39.11)
	One sexual partner	210 (55.12)
	≥2 sexual partner	22 (5.77)
Duration (in years)	≤ 5	86 (22.57)
	>5	295 (77.43)
WHO clinical stage	I	195 (51.18)
	II	74 (19.42)
	III/IV	112 (29.40)
Recent viral load	Undetectable	361 (94.75)
	Detectable	20 (5.25)
Baseline CD4 count	< 500 cell/mm^3^	315 (82.68)
	≥500 cell/mm^3^	66 (17.32)
Regimen Change	Yes	335 (87.93)
	No	46 (12.07)
History of TB co-infection	Yes	24 (6.30)
	No	357 (93.70)
Missed clinical appointment	Yes	75 (19.69)
	No	306 (80.31)
Disclosure status	Yes	345 (90.55)
	No	36 (9.45)
Social support	Poor	195 (51.18)
	Intermediate	149 (39.11)
	Strong	37 (9.71)

^*^Pastoralist, retired, self-employed, NGO, driver, unemployed, evangelist.

### Uptake of community ART model

**Figure 2:**
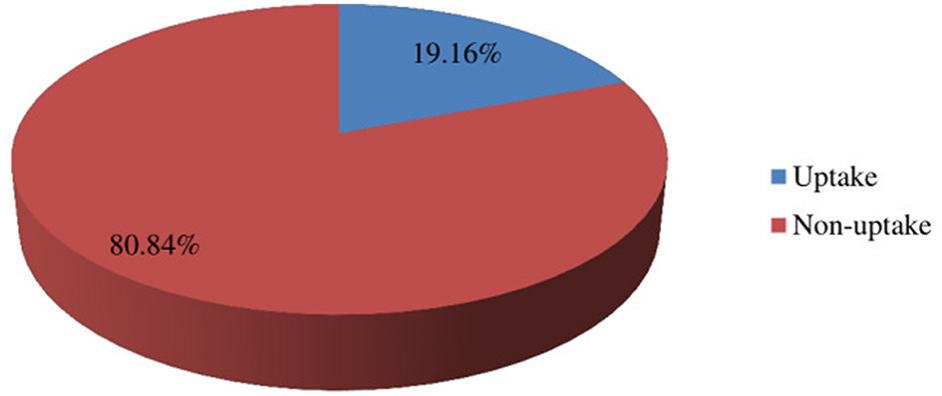
The uptake of community-based DSD models among people living with PLHIV in public health facilities in South Ethiopia, 2023 (*n* = 381).

The uptake of the community-based ART DSD model was 19.16% (95% CI: 15.19%, 23.13%); (Figure 2).

### Factors associated with community ART model uptake

Residence, educational status, occupational status, number of sexual partners, duration of ART, and disclosure were the factors associated with the uptake of community-based ART DSD models in the bivariable logistic regression analysis. The multivariable logistic regression analysis showed that residence and disclosure with were significantly associated factors with the uptake of the community-based models among PLHIV, with a *p*-value of < 0.05.

The odds of uptake of the community-based ART DSD models for patients in urban areas were two times higher than those of patients in rural areas (AOR 2.30, 95% CI: 1.12, 4.70). Additionally, patients who disclosed their HIV status were 4.44 times more likely to utilize the community-based ART DSD models compared to those who never disclosed their HIV status (AOR: 4.44, 95% CI: 1.04, 18.97); (Table 2).

**Table 2:** Bivariable and multivariable logistic regression analyses for factors associated with the uptake of community-based DSD models among PLHIV in public health facilities of South Ethiopia, 2023 (*n* = 381).

**Variables**	**Community-based DSD model uptake**		**COR (95% CI)**	**AOR (95% CI)**	***p*-value**
	**No (%)**	**Yes (%)**			
**Residence**					
Urban	226 (78.20)	63 (21.80)	2.29 (1.12, 4.67)	2.30 (1.12, 4.70)	0.023
Rural	82 (89.13)	10 (10.87)	Reference	Reference	
**Education**					
No formal	77 (82.80)	16 (17.20)	Reference	Reference	
Primary	115 (80.99)	27 (19.01)	1.13 (0.57, 2.24)	1.03 (0.48, 2.13)	0.939
Secondary	73 (75.26)	24 (24.74)	1.58 (0.78, 3.21)	1.99 (0.85, 4.66)	0.111
Tertiary	43 (87.76)	6 (12.24)	0.67 (0.24, 1.84)	0.76 (0.22, 2.65)	0.663
**Occupation**					
Daily laborer	51 (80.95)	12 (19.05)	2.06 (0.61, 6.91)	1.46 (0.41, 5.23)	0.562
Merchant	70 (84.34)	13 (15.66)	1.63 (0.49, 5.35)	1.07 (0.31, 3.77)	0.911
Government employee	67 (81.71)	15 (18.29)	1.96 (0.60, 6.35)	1.25 (0.36, 4.38)	0.729
Housewife	63 (73.26)	23 (26.74)	3.19 (1.02, 9.98)	2.07 (0.63, 6.86)	0.232
Farmer/student	35 (89.74)	4 (10.26)	Reference	Reference	
Other	22 (78.57)	6 (21.43)	2.39 (0.60, 9.42)	1.68 (0.39, 7.15)	0.484
**Catchment of ART facility**					
Within	232 (78.38)	64 (21.62)	2.33 (1.11, 4.90)	1.67 (0.69, 4.09)	0.258
Outside	76 (89.41)	9 (10.59)	Reference	Reference	
**Number of sexual partners**					
No sexual partner	124 (83.22)	25 (16.78)	4.23 (0.54, 32.94)	3.08 (0.37, 25.37)	0.295
One sexual partner	163 (77.62)	47 (22.38)	6.06 (0.79, 46.20)	4.93 (0.61, 39.60)	0.133
≥2 sexual partner	21 (95.45)	1 (4.55)	Reference	Reference	
**Disclosure**					
Yes	274 (79.42)	71 (20.58)	4.41 (1.03, 18.78)	4.44 (1.04, 18.97)	0.045
No	34 (94.44)	2 (5.56)	Reference	Reference	
**Duration on ART**					
≤ 5	65 (75.58)	21 (24.42)	Reference	Reference	
>5	243 (82.37)	52 (17.63)	0.66 (0.37, 1.18)	0.62 (0.34, 1.11)	0.109

## Discussion

This study aimed to explore the uptake and associated factors of community-based DSD models among PLHIV. The uptake of the community-based DSD models was nearly 20%. Residence and serostatus disclosure were statistically significant factors associated with the uptake. These findings indicate that the uptake of community-based DSD models requires attention from policymakers and program planners. Efforts should be made to address the barriers faced by stable PLHIV on ART to enhance the uptake of these models, which are crucial for improving adherence, retention in care, and decongesting healthcare facilities.

Our study showed that the proportion of uptake of the community-based DSD model was 19.16%. This finding is lower than that of studies conducted in KwaZulu-Natal, South Africa (16) and Durban, South Africa (15). The sample size, study design, and participants' eligibility criteria could explain this variation. The study conducted in Durban, South Africa, used a randomized controlled trial design. Another possible explanation might be that PLHIV on ART in our setting might prefer a facility-based DSD model over community-based DSD models. These findings have significant implications for decreasing the burden on health facilities by promoting the community-based DSD model (31).

However, our study's findings are higher than those from previous studies conducted in Malawi (14), Kampala, Uganda (10), Mulago, Uganda (12), and Arua district, Uganda (13). The difference might be due to study setting variations, health service delivery systems, and eligibility criteria, and the definition used for the outcome could also contribute to the differences observed. In our study, the uptake of community-based DSD models refers to the uptake of either community ART groups (CAG) or peer lead community ART distribution (PCAD), whereas studies conducted in Malawi and Uganda indicate the uptake of CAG.

The findings of this study revealed that urban residents living with HIV are more likely to uptake community-based DSD models than rural residents. The possible explanation might be the better socioeconomic status, good adherence, and disclosure status of clients in urban areas than those in rural areas. A systematic review and meta-analysis revealed that clients living in urban areas have better adherence than those living in rural areas (32). However, this study finding is inconsistent with early programmatic data from Zimbabwe, which indicates that the uptake of community-based DSD models is highest in rural areas (33). This result suggests that urban resident clients are more likely to utilize community-based DSD models. Further studies are needed to identify the association between the place of residence and the uptake of community-based DSD models.

Furthermore, in this study, HIV-positive patients who disclosed their status were significantly more likely to uptake community-based DSD models. The reason might be that patients who disclose their HIV status may have strong social support and better adherence to ART services. Serostatus disclosure of HIV is significantly associated with better engagement in medical care, reduced HIV transmission, improved ART adherence, decreased psychological distress, and enhanced social support opportunities (34). PLHIV who disclosed their HIV status demonstrated better adherence than those who did not (35). In addition, the community-based DSD model may increase the risk of serostatus disclosure, which poses a significant challenge to the uptake of these models (36, 37). This finding suggests that disclosing HIV serostatus enhances the uptake of community-based DSD models.

### Strengths and limitations of the study

The study was conducted at multicenter public health facilities, enhancing its generalizability. However, due to the cross-sectional study design, the study's limitations include the difficulty in establishing cause-and-effect relationships.

## Conclusion

One out of five PLHIV utilizes the community-based DSD models. Urban residence and serostatus HIV disclosure were significantly associated with the uptake of community-based DSD models. Increasing the uptake of community-based DSD models might improve ART outcomes and the efficiency of the healthcare system by improving adherence, viral suppression, and retention in care and service delivery efficiency among PLHIV. Therefore, to increase adherence and retention in ART, efforts should be made to improve the uptake of community-based DSD models by encouraging HIV serostatus disclosure. Improvement in uptake is essential for the resource-limited healthcare system of Ethiopia.

## Data availability statement

The original contributions presented in the study are included in the article/supplementary material, further inquiries can be directed to the corresponding author.

## Ethics statement

The studies involving humans were approved by Institutional Review Board of Arba Minch College of Health Science. The studies were conducted in accordance with the local legislation and institutional requirements. The participants provided their written informed consent to participate in this study.

## Author contributions

FM: Conceptualization, Data curation, Formal analysis, Funding acquisition, Investigation, Methodology, Project administration, Resources, Software, Supervision, Validation, Visualization, Writing – original draft, Writing – review & editing. TT: Methodology, Resources, Supervision, Validation, Visualization, Writing – review & editing. AA: Project administration, Supervision, Validation, Visualization, Writing – review & editing. TG: Data curation, Formal analysis, Investigation, Methodology, Resources, Software, Supervision, Validation, Visualization, Writing – review & editing.
